# Developing evidence briefs for policy: a qualitative case study comparing the process of using a guidance-contextualization workbook in Peru and Uganda

**DOI:** 10.1186/s12961-019-0488-0

**Published:** 2019-11-21

**Authors:** Elizabeth Alvarez, John N. Lavis, Melissa Brouwers, Gloria Carmona Clavijo, Nelson Sewankambo, Lely Solari, Lisa Schwartz

**Affiliations:** 1 Department of Health Research Methods, Evidence and Impact (HEI), Faculty of Health Sciences, McMaster University, CRL 2nd Floor, 1280 Main Street West, Hamilton, ON L8S 4K1 Canada; 2 Centre for Health Economics and Policy Analysis (CHEPA), McMaster University, Hamilton, ON Canada; 3 McMaster Health Forum, McMaster University, Hamilton, ON Canada; 4 School of Epidemiology and Public Health, University of Ottawa, Ottawa, ON Canada; 5 Instituto Nacional de Salud (INS), Ministry of Health, Lima, Peru; 6 Makerere University College of Health Sciences, Kampala, Uganda

**Keywords:** Comparative, evidence brief, context, knowledge translation, health systems strengthening, qualitative case study, maternal and newborn health

## Abstract

**Background:**

Translating research evidence from global guidance into policy can help strengthen health systems. A workbook was developed to support the contextualization of the WHO’s ‘Optimizing health worker roles to improve maternal and newborn health’ (OptimizeMNH) guidance. This study evaluated the use of the workbook for the development of evidence briefs in two countries — Peru and Uganda. Findings surrounding contextual factors, steps in the process and evaluation of the workbook are presented.

**Methods:**

A qualitative embedded case study was used. The case was the process of using the workbook to support the contextualization of global health systems guidance, with local evidence, to develop evidence briefs. Criterion sampling was used to select the countries, participants for interviews and documents included in the study. A template-organizing style and constant comparison were used for data analysis.

**Results:**

A total of 19 participant-observation sessions and 8 interviews were conducted, and 50 documents were reviewed. Contextual factors, including the cadres, or groups, of health workers available in each country, the way the problem and its causes were framed, potential policy options to address the problem, and implementation considerations for these policy options, varied substantially between Peru and Uganda. However, many similarities were found in the process of using the workbook. Overall, the workbook was viewed positively and participants in both countries would use it again for other topics.

**Conclusions:**

Organizations that produce global guidance, such as WHO, need to consider institutionalizing the application of the workbook into their guidance development processes to help users at the national/subnational level create actionable and context-relevant policies. Feedback mechanisms also need to be established so that the evidence briefs and health policies arising from global guidance are tracked and the findings coming out of such guideline contextualization processes can be taken into consideration during future guidance development and research priority-setting.

## Introduction

Weak health systems hinder the delivery of and access to effective clinical and public health interventions to those most in need, especially in low- and middle-income countries, where these systems can be over-burdened and under-resourced [1–3]. Evidence-based health systems guidance at a global level is one way to support countries facing the same or similar health challenges (for example, lack of trained health workers) to respond with an adequate approach [3]. Health systems guidance has been defined as “*systematically developed statements produced at global or national levels to assist decisions about appropriate options for addressing a health systems challenge in a range of settings and to assist with the implementation of these options and their monitoring and evaluation*” [3]. Global guidance can, in turn, be used to (1) develop policies at the global level (for example, funding vertical or single-disease programmes vs. integrated care); (2) develop guidance at the national level (for example, Evidence-Informed Policy Network (EVIPNet) developing an evidence brief); and (3) develop policies at the national or subnational level (for example, Ministry of Health policy-making at the national level) [4].

In order for guidance to have an impact, the issue first needs to get on a government’s agenda, guidance needs to be contextualized or adapted to the particular setting and inform policy development, and a policy needs to be approved and implemented [4, 5]. Until recently, to our knowledge, there was no tool available to support users of health systems guidance in combining global recommendations with local assessments of the problem and its causes, existing health systems arrangements (delivery, financial and governance arrangements) and political system considerations (institutions, interests, ideas and external factors) in order to help with the contextualization process [4]. Considering these factors during policy development can ensure the policy options are designed to meet the needs and realities of a given setting, which can then aid with implementation [4].

A workbook for contextualizing health systems guidance (henceforth, ‘workbook’) was created to support the contextualization of a WHO guidance document on optimizing health worker roles for increasing access to and use of key interventions for improving maternal and newborn health in low- and middle-income countries (OptimizeMNH guidance) through the development of an evidence brief [6]. A description of the steps and critical factors in the development process were published in this journal [7]. An evidence brief is a document that brings together different types of research evidence to address what is known about a problem, possible ways to address the problem, and implementation considerations. Evidence briefs can then be used to inform policy dialogues, where policy-makers, researchers and stakeholders discuss these issues and can inform policy through a dialogue summary [8].

The workbook followed a newly developed framework called the ‘health systems guidance contextualization framework’, which addressed (1) clarifying the problem and its causes; (2) framing options for addressing the problem; (3) identifying implementation considerations; (4) considering the broader health system context; (5) considering the broader political system context; (6) refining the statement of the problem, options and implementation considerations in light of health system and political system factors; (7) anticipating monitoring and evaluation needs; and, lastly, (8) making national or subnational policy recommendations or decisions [9]. Briefly, the workbook provides questions to guide users through each of the eight steps in the health systems guidance contextualization framework, gives examples related to the topic, and prompts users for type(s) of evidence to answer each question (for example, systematic reviews, local studies, administrative data).

Given that this was a new tool, we sought to examine whether and how it could be used in the process of contextualizing health systems guidance. This study also allowed comparisons to be made between the use of the guidance-contextualization workbook in two settings, namely Peru and Uganda, along with their distinct contextual factors. Lastly, findings from this study were used to update the workbook. An updated version of this workbook can be found at https://www.mcmasterforum.org/lets-collaborate/resources [10].

## Methods

### Study design

This study followed an embedded single case study design [11] that incorporated two units of analysis, that is, two countries. The case was the process of using the workbook to support the contextualization of global health systems guidance, with local evidence, to develop evidence briefs [4, 8, 9, 11, 12]. Local evidence can include regional, national, provincial/state or municipal data. The case was bounded by setting or country, participants (policy-makers, stakeholders and researchers) and time, starting with the countries’ decision to use the workbook through the development of draft evidence briefs [11]. Because multiple people were involved in the process of using the workbook, a constructivist paradigm was fitting for this study [11, 13].

### Sampling and recruitment

Criterion sampling [14] was used to select the two countries for this study. The criteria included (1) commitment from policy-makers, the WHO country office, regional office and/or headquarters, or from the Ministry of Health to support the development of an evidence brief by using the workbook to contextualize the OptimizeMNH guidance, and (2) the prerequisite that the country had not conducted formal work on task shifting with regards to the OptimizeMNH guidance.

Criterion sampling, based on involvement in the case, was used to select participants for semi-structured interviews in each country. Recruitment was conducted through personalized emails. In addition, criterion sampling was used to select documents related to the use of the workbook (for example, meeting minutes) or information about the contexts in which the workbook was used (for example, Uganda health assessment).

### Data collection

Several data collection techniques were used. Participant-observations were conducted with the country teams in Peru and Uganda, and semi-structured interviews were conducted over Skype or email – each by one member of the team (EA). As a participant-observer, EA participated in all aspects of using the workbook with the country teams, including developing the workflow for the project, determining the composition of the teams, working through the workbook, gathering evidence and developing the evidence briefs. This work was also supported by JL, an expert in health systems and policy research. While these roles entailed providing support for the work (for example, finding peer-reviewed literature), ultimately, key decisions were made by the country teams in order to observe real-life processes in developing evidence briefs. The interviews were recorded with a digital recorder and transcribed (or translated and summarized into English for interviews conducted in Spanish). Field notes were made during and after the interviews. The interviewer asked participants about their role in, the process of, and their impressions of using the workbook, including useful components and areas to be improved. Because of the interplay between the researcher and data collection in qualitative research, a reflexive journal was kept [15]. Journal entries, emails, field notes, meeting notes and documents were all used as data (Additional file 1).

### Data analysis

A comparative approach was used throughout the analysis in order to highlight major points of difference in the process of using the workbook between Peru and Uganda, major issues arising from the application of the workbook, and insights for future work. An initial guiding code structure was created and codes were added or modified throughout the analysis as guided by the data. Following a template-organizing style and constant comparison, data were coded and organized by a single reviewer (EA) into a table using Microsoft Word. Notes were made throughout to keep track of emerging concepts and these were then linked into themes [15]. Once themes were developed, all of the data were once again reviewed to make sure no new concepts arose and no rival theories were found. As all participants were researchers, they were all asked to review the manuscript as part of the peer debriefing process.

## Results

### Data collected

A total of 19 meetings (participant-observations) were conducted (4 with participants in Peru and 15 with participants in Uganda). Interviews were conducted with 8 participants (3 in Peru and 5 in Uganda), including all potential participants who were involved with the country teams in developing the evidence briefs using the workbook. For more detailed information about the country teams and usual processes for development of evidence briefs in Peru and Uganda, please see Additional file 2. One interview was conducted with each participant over Skype, except for one interview conducted through email. These interviews ranged from 31 to 85 min in length, with an average duration of 59 min. While specifics of the participants are not provided for confidentiality reasons, it is worth noting that the participants were diverse in age, gender and level of seniority in their respective positions. All people interviewed had a background as researchers. All but one interviewee also had a background as health professionals and/or policy-makers. In addition, 50 documents were reviewed (not including the documents reviewed for developing the evidence briefs in either country). Multiple personal emails and a reflexive journal, which included the field notes, were also used as data.

### Select contextual features in Peru and Uganda

Every step of the workbook prompted users to consider data from their own contexts in the development of the evidence brief. This was seen by participants as useful since it served as a checklist for the types of information that were needed. Documents and information from the meetings were used to gather information about the contexts in Peru and Uganda. These countries varied significantly in their geographic, social and demographic factors, in their health systems arrangements, and in their health indicators. Additional file 2 provides many of these descriptions, so only a few points will be highlighted here. First, both countries had a centralized health policy authority yet systems for management and implementation of health policies and services were decentralized (divided by regions in Peru and by districts in Uganda). While Peru is about five times larger geographically than Uganda, Uganda had four times more administrative units than Peru, which may have increased the local capacity to self-govern and to adapt policy and services to the communities’ needs, but also required more human and financial resources [16–19]. Additionally, while more than three-quarters of Peruvians lived in urban areas, fewer than one-fifth of Ugandans lived in urban centres, which may have had a significant impact on the delivery of services [20, 21]. Both Peru and Uganda had a mix of public, private, and donor funding and delivery of health services, but the contribution of each of these was quite different in both countries. One example is that donor financing in Peru accounted for 2% of the total health expenditure, while in Uganda, donor financing ranged from 32% to over 50% [22, 23]. One common problem in both health systems was the minimal cooperation between the public and private systems, which can lead to poor planning and coordination [22, 23].

Issues surrounding maternal and child mortality are complex, and multiple factors need to be considered to ensure appropriate policy options are implemented. As noted by the participants and in journal entries, the workbook helped users identify some of these considerations. Step 2 of the workbook asks users to identify available health cadres in their setting. Table 1 illustrates the complexity of contextualizing global guidance to a specific setting, as health cadres go by different names and have varying levels of training in different settings. These differences were important in developing the options and determining implementation considerations. For example, many interventions listed for lay health workers required some level of biomedical training, which community health workers in Uganda generally lacked. It was important to have health workers who were familiar with the health cadres, and the interventions performed by each health cadre, to elicit this level of information.

**Table 1:** Health cadres addressed by WHO’s OptimizeMNH guidance and their equivalents in Peru and Uganda

Health care cadres – WHO	Peru	Uganda
Non-specialist doctor	General surgeons (Médicos cirujanos generales) Family doctors (Médicos de família) General doctor not certified in voluntary surgical contraception or sterilization/vasectomy (Medico general no calificado en anticoncepción quirúrgica voluntaria) Doctors certified in the provision of contraceptives, including voluntary surgical contraception or sterilization/vasectomy (Médicos calificados en provisión de anticonceptivos incluido anticoncepción quirúrgica voluntaria)	Medical officer
Advanced level associate clinician	Did not exist in Peru	Did not exist in Uganda
Associate clinician	Did not exist in Peru	Clinical officer
Midwife	Midwife (Obstetriz)	Midwife; there was some cross-over with nurses (see below). They had 2 levels – enrolled and registered (see below)
Nurse	Nurse (Enfermero(a))	Had 2 main levels of nurses: - enrolled nurses – certificate - registered nurses – diploma (more training than enrolled nurses) Other levels: - nurses with Bachelor’s degree - ‘comprehensive’ or ‘double-trained’ nurse with training in both nursing and midwifery
Auxiliary nurse	Nurse technician (Técnico de enfermería)	Uganda used to have nurse assistants, but they were no longer being trained or recruited; they still practiced but were no longer officially recognized; it would take time to train this cadre again as training programmes had stopped
Auxiliary nurse midwife	Did not exist in Peru	Uganda used to have this cadre as part of the auxiliary nursing group but they were no longer recognized
Lay health worker	Health promoter (Promotores de salud)	Community health worker; however, in Uganda, they tended to be volunteers in the community and may not have had any health training or have been literate

The knowledge translation processes in Peru and Uganda varied significantly in their structures (Additional file 2). Most notably, Peru’s process for developing evidence briefs included fewer people and the outcome of the evidence briefs and further policy dialogues could lead to policy decisions implemented directly by the Ministry of Health. In Uganda, more people were involved in developing the evidence brief, including a Secretariat in charge of gathering evidence and writing the document, and a working group to provide input. This participatory process is relevant, as the evidence brief and policy dialogue serve to inform the policy process, which involves the Ministry of Health along with other Ministries and donor organizations. Health policy in Uganda is ultimately approved by the Cabinet.

### Main findings in the process of using the workbook to develop evidence briefs in Peru and Uganda

Key steps found in the process of contextualizing the OptimizeMNH guidance included (1) selecting the topic; (2) identifying the venue (decision-making authority such as national or subnational); (3) clarifying the problem; (4) framing the policy options; (5) identifying implementation considerations; (6) identifying equity considerations; (7) considering the broader health system context; (8) considering the broader political system context; (9) refining the statement of the problem, options and implementation considerations in light of health systems and political system factors; (10) anticipating monitoring and evaluation needs; and (11) making national policy recommendations or decisions by developing the evidence brief, planning for a policy dialogue and engaging the public. A summary of the main findings for each key step, along with examples, are provided below for those looking to use the workbook in their settings.

In both countries, the workbook was seen as a methodology for developing the evidence briefs. In Uganda, the workbook also helped define the overall process by refining the structure of the terms of reference for development of the evidence brief and by creating a timeline.

#### Selecting the topic

The workbook did not address ‘selecting a topic’ as an explicit step. However, it was found that selecting the topic was a discrete step in the process, even with an available guidance document. Factors for selecting the topic included (1) alignment with priorities of the government or Ministry of Health, (2) alignment with the OptimizeMNH guidance, which included taking a step back to evaluate whether the guidance addressed the cause of the problem for that setting, (3) consideration of priority regions/populations, and (4) preliminary consideration of the relevant OptimizeMNH recommendations to ensure there was enough substance for developing an evidence brief on that topic. The topic was narrowed down to increasing access to modern family planning methods in Loreto region, Peru, because this was perceived as an important issue to tackle by the Ministry of Health. In Uganda, the topic remained aligned with the full guidance on optimizing health worker roles to increase access to and use of key interventions to improve maternal and newborn health.

#### Identifying the venue

The workbook mentioned that a venue for decision-making should be identified (Step 8 in the workbook) to determine the audience, format and language for presenting the evidence. It was found that this step was more prominent and discrete, yet iterative, in relation to selecting the topic and other aspects of developing the evidence brief. For example, deciding whether the target venue was at the national or subnational level was discussed for both countries, following the suggestions of the workbook. The venue turned out to be different based on the contextual factors of each country. Determining the venue was important for deciding who to involve in the process and the context used for clarifying the problem, framing the options and identifying implementation considerations, including consideration of health system and political system contextual factors.

The venue for Peru was chosen as the health authority at the regional level (Loreto) and for Uganda it was the Ministry of Health at the national level. Several factors were considered in selecting the venue for each country, including (1) the level of government responsible for health policy and/or implementation, (2) the government’s commitment to evidence-informed policy-making, (3) established professional connections, (4) types of research evidence available within the country (national vs. regional level data), and (5) other considerations such as prior laws or the level of authority for regulations and training of health workers.

#### Clarifying the problem

In this step (Step 1 in the workbook), the OptimizeMNH guidance and the workbook clarified the problem in several ways. First, WHO had already narrowed the topic to some extent, found the evidence and made recommendations. Second, questions in the workbook guided users in breaking down considerations of potential contributors to the problem for a particular setting. Third, the workbook provided examples of how to use the guidance to answer these questions.

Using the workbook identified gaps in policy and in existing evidence. For example, it was found that many relevant past policies in both countries had not been fully implemented. Working with a team also helped clarify the problem. For example, in Peru, country experts knew that framing the topic in line with the Millennium Development Goals (MDGs) was not as useful as framing it within country goals because of the importance of aligning the definition of the problem with priorities of the Ministry of Health.

“*The guidance is geared towards the MDGs but these are up next year, but the relevance is not in the MDGs, but the relevance of family planning falls within an objective of a national health strategy that extends until 2020, so within that framing it remains very important*” (Member of Secretariat in Peru, Interview 2–01)

#### Framing the policy options

The second step in the workbook was to frame the policy options. After determining available health cadres in either country, each applicable recommendation was worked through to assess whether the cadre was performing the specific intervention. If not, and the OptimizeMNH guidance suggested it could be safe to do so, then the Secretariat discussed what it would take to be able to have the cadre provide that intervention and what supports would be needed to do so. In addition, the workbook asked users to address the potential benefits, harms and costs associated with each possible option. Considering other factors, such as feasibility, impact of the proposed changes and scope of the OptimizeMNH guidance, helped determine which options to develop. Peru developed three possible policy options and Uganda developed five policy options for their draft documents.

Lastly, conducting key informant interviews with health workers in the country confirmed the appropriateness of the options developed by the Secretariat in Uganda. The decision to carry out this step was not linked to the workbook but rather to the will of the Secretariat to ensure its recommendations were accurate and reflected the actual practice of the various health cadres in the country.

#### Identifying implementation considerations

The workbook asked users to identify the potential barriers to implementation at the level of healthcare recipients, healthcare providers, organizations and systems, and asked about strategies to overcome these barriers. Discussions at meetings around clarifying the problem and considering the reasons why past policies had not been implemented helped identify implementation considerations. In addition, brainstorming while developing the policy options and using the OptimizeMNH guidance and other research evidence identified further implementation considerations. For example, the OptimizeMNH guidance provided general implementation considerations that had been found through the development of the recommendations, and targeted searches found cadre-specific or country-specific barriers and facilitators.

#### Identifying equity considerations

Questions surrounding issues of equity were asked at the end of Steps 1, 2 and 3 in the workbook as they related to clarifying the problem, framing the options and identifying implementation considerations. Because of the venue and topic selection process, equity was a focus in Peru throughout the development of the evidence brief by bringing attention to an area of high need (Loreto), as revealed through local data. In Uganda, the topic was more general and equity considerations were specifically addressed by the workbook, which may have been missed without these prompts. Brainstorming and using evidence identified high-risk groups in Uganda.

#### Considering the broader health system context

The workbook prompted users to consider health system factors, including delivery, financial and governance arrangements. In addition, specific considerations were provided for each health system arrangement. Several participants noted that these aspects of the workbook were very helpful and easy to understand, even for users without a health systems and policy background. Generally, discussions of the health system context during meetings aided in (1) determining the policy venue for the work, (2) developing the options and (3) identifying implementation considerations. One example of this was that, in Uganda, discussions around delivery arrangements included what facilities were located where (for example, health centre II vs. hospitals) and who staffed these facilities. These discussions were especially helpful in focusing the options on where health professionals would be practicing on their own, and therefore where these intervention/cadre combinations were most needed. In addition, barriers at these levels were discussed.

#### Considering the broader political system context

The workbook also provided political system factors, including institutions, interests, ideas and external factors, and broke down components of each. This was seen as helpful by the participants in that people without health systems and policy backgrounds would be able to understand the terms used. Discussions of the political system context during meetings helped (1) frame the problem, (2) develop the options and (3) identify implementation considerations. For example, during the development of the evidence brief, there was a high rate of turnover in administration in Peru, making this part of the assessment difficult. However, more stable parts of the political system, such as prominent ideas (e.g. beliefs held in government and society) and past policies (an example of an institutional factor), played a role in defining the problem. In Uganda, it was noted that the role of non-governmental organizations and/or donors would need to be taken into consideration in the policy decision-making process.

#### Refining the statement of the problem, options and implementation considerations considering health system and political system factors

The workbook provided an area to write down a summary of findings from each of the above steps in order to refine the statement of the problem, options and implementation considerations. In both countries, this occurred in an iterative manner throughout the process. As one part was developed, another would be modified based on the findings (for example, problem definition in Peru based on political system considerations). Thus, this section served as a reminder to review all of the work, as in a checklist.

#### Anticipating monitoring and evaluation needs

Step 7 of the workbook addressed monitoring and evaluation (M&E) for each option. M&E were not addressed explicitly in Peru. Monitoring systems were already in place, including national surveys such as the Encuesta Demográfica y de Salud Familiar (ENDES or Demographic and Family Health Survey) and Encuesta a Establecimientos de Salud con Funciones Obstétricas y Neonatales (ENESA or Survey of Health Facilities Providing Obstetric and Neonatal Care). Even though gaps were found, these two national surveys provided much information related to the problem at hand and were expected to be conducted on a routine basis. In Uganda, this section was addressed in general terms (for example, acceptability, effectiveness, numbers performed), where a section for M&E had been included for each option. It is expected that M&E for each option will be developed further in both countries as more experts provide feedback on the draft documents.

#### Making national policy recommendations or decisions by developing the evidence brief, planning for a policy dialogue and engaging the public

The workbook provided information on how to go about developing an evidence brief, planning for a policy dialogue and engaging the public, where applicable. This section of the workbook was not used because both teams already had experience developing evidence briefs. This section would likely be more useful for teams that have not produced evidence briefs or conducted policy dialogues in the past. In addition, involving the public has not been discussed thus far in either country; this will likely not occur until more policy-makers and stakeholders are involved in policy dialogues.

### Evaluating the process of using the workbook in Peru and Uganda

Several benefits and limitations in the process of using the workbook for developing evidence briefs were expressed by participants in both countries. A summary of these findings is provided below. It is important to note, however, that these comments reflect three changes to their usual processes of developing evidence briefs, namely (1) a workbook, which was specific to contextualizing the OptimizeMNH guidance, was provided, (2) two authors of the workbook (and of this study) provided support throughout the process as participant-observers, and (3) the OptimizeMNH guidance was worked through systematically. According to country experts, guideline recommendations from WHO were not generally applied systematically but were used as a reference at the national level and in unknown ways for subnational levels. For example, if a priority topic aligned with WHO guidance, then the recommendations were used as one input. However, in this study, each relevant recommendation was worked through, in turn. One participant speculated that the reason for the guidance being used systematically in this case was due to this being part of a research study and paying more attention to the details of using the workbook. Another participant added that it was not so much a change in the process but, rather, that the workbook helped take account of specific contextualization items such as defining the scope, looking for local figures, analysing the available resources (when defining implementation issues) and other aspects that the usual methodology for developing evidence briefs did not stress as much as the workbook. Overall, users of the workbook seemed pleased with the workbook and the process of using it, and several people in both Peru and Uganda stated that they would use (or are already using) the workbook for other projects.

“*I am using it. For another evidence brief …I am using the workbook to help me gather the information I need.*” (Member of Secretariat in Peru, Interview 2–01)

*“I definitely think so. I mean, if a question came to me I do not know why I would suffer going through the usual stuff that we do, when the workbook is over here, there’s no way.* [laughter] *There’s no way.*” (Member of Secretariat in Uganda, Interview 2–04)

#### Benefits and limitations

1. The workbook, which was specific for contextualizing the OptimizeMNH guidance and provided examples linking the questions to the guidance, made the process faster and easier when compared with prior processes.

“*Because you know, with the SURE guides, you have a guide and then you know, it’s like a map … to navigate your way through a forest – yeah. And with the workbook, I mean there are several roads through the forest, and so you choose depending on which of those would suit you or not, so it makes things a bit easier*.” (Member of Secretariat in Uganda, Interview 2–05)

Even though it was felt the workbook was dense, or at times ‘tedious’, to work through because of the large number of sections and questions asked, no specific modifications were recommended by participants when asked directly which sections could be left out or cut down.

2) The structure of the workbook was seen as systematic, logical and user-friendly for developing evidence briefs but also served as a checklist for evaluating the work. There was a potential concern raised by one interviewee that, by including so many considerations and having it be so systematic, it might paradoxically limit peoples’ thinking about other considerations not listed in the workbook.
3) The workbook was easy to understand and the terms were well described, which facilitated the contextualization process. There was a potential concern raised by participants that the policy language and English terms used in the workbook might be difficult for those with no training in health systems and policy or non-native English speakers to understand. For example, there were some English concepts that could not be translated into Spanish or were not exactly the same when translated.
4) The workbook had a missing component — a glossary of medical terms. Some medical terms in the OptimizeMNH guidance were not described. Interviewees expressed that a glossary of these terms, either in the guidance document or in the accompanying workbook, would be useful for non-health professionals. Working with teams involving health workers bypassed this problem during the study.
5) Prompts and examples in the workbook were valuable for the contextualization process. In particular, examples of which types of evidence could be used to answer various questions (for example, about the problem) were especially helpful for those not familiar with developing evidence briefs. Prompts also highlighted gaps in knowledge and in practice. One example is that, in Uganda, only 38% of urban and 29% of rural births were registered from 2005 to 2012, which made the Secretariat unsure of how to interpret other findings for Uganda.

“*Gaps in knowledge were found in Uganda, with discussion of cadres/interventions/barriers (for example, where do women die in Uganda and from what?)*” (Participant-observation in Uganda, June 13, 2014)

There was disagreement over the need for more examples for those in areas with limited data (for example, countries without a routine national demographic survey) or for those without training in health systems and policy.

6) Participants felt that, although costs were addressed in the workbook, they needed to come out more explicitly in the evidence brief since policy-makers are generally very interested in costs.
7) Outside of the workbook, country experts were needed for both content and methods in developing evidence briefs. Both countries had a mix of health workers on their teams. They understood the medical terminology used in the OptimizeMNH guidance and their respective health systems, and they had knowledge of available cadres and interventions performed by each cadre for developing the options and identifying implementation considerations. Having methodological expertise was also necessary, as evidence briefs draw on quantitative as well as qualitative data.
8) Alongside the workbook itself, outside support built capacity and focused attention on the work. In Peru, outside support assisted with the capacity to find evidence and to develop the evidence brief. In Uganda, everyone who worked on this project was volunteering their time and had many other priorities. Having outside support brought focus to this work.
9) The process of using the workbook also evaluated the OptimizeMNH guidance and standardized thinking. The use and implementation of WHO guidelines are not usually evaluated. Participants felt the guidance was unclear about what countries could do when their health cadres or recommendations did not align with the information presented. According to participants, working through the guidance would lead to policy-makers looking beyond their contexts and standardize their thinking with what is happening at a global level.

“*… so if you’re talking about lay health workers, what exactly does this mean in your context and also in the outside context…*” (Member of Secretariat in Uganda, Interview 2–04)

## Discussion

### Principal findings

Overall, the workbook was seen as useful by participants in both countries. Several interviewees stated that they would use (or are already using) the workbook for developing other evidence briefs. Even though the workbook was developed with examples to help contextualize a particular guidance document, it is also generic enough to be used for other topics. Several benefits and limitations were found in the process of using the workbook, keeping in mind that the usual processes were also changed by having outside support and by systematically working through the OptimizeMNH guidance.

As can be seen, the way the problem and its causes are framed, potential policy options to address the problem and its causes, and implementation considerations for these policy options varied substantially between Peru and Uganda due to their contextual factors. For example, in selecting a topic, the starting point for both countries was the OptimizeMNH guidance, but because of priorities of the Ministry of Health in Peru, the work became narrowed down to specific interventions around family planning in an area of high need (Loreto), while in Uganda, government priorities allowed for the topic to remain broad. Further, the cadres that were available in each country, and the interventions performed by these cadres, varied significantly. Finally, implementation considerations differed in each country based on epidemiological features (for example, prevalence of HIV in Uganda), past policies (for example, sterilizations of the poor in Peru), and other issue- and context-specific factors. This case is one example of how and why contextualization of guidance is important.

### Strengths and limitations of the study

The case study design, including multiple sources of data, allowed for an in-depth examination of the process and context of using the workbook, both at an international level (for example, global guidance around improving access to and use of key interventions to improve maternal and newborn health) and at a national or sub-national level (for example, health systems arrangements within each country or region). Studying the process of using the workbook in two countries allowed for comparisons between key findings in these different settings. Lastly, the roles of the authors as participant-observers added to the richness of the findings, and peer debriefing refined the concepts and themes.

The main challenge is that the workbook is used for a defined purpose and time in the development of evidence briefs. This means that the rest of the process of convening policy dialogues, developing policy that is informed by the evidence brief and policy dialogue, implementing that policy, and evaluating the impact of the policy on the population’s health is outside the scope of this study. Therefore, how the contextual factors and components of the processes found in this study affect the ultimate policy decisions and their implementation is not known. For example, is it better for a country to have an EVIPNet team with direct organizational ties to the Ministry of Health, such as in Peru, or is it better to have a broader participatory process such as in Uganda? The implications of this and other differences found need further examination to determine the strengths and limitations of country level mechanisms. While some of the findings are transferable to other contexts, one limitation of a case study approach is that findings cannot be generalized from these to other cases. Therefore, readers would need to consider their own contexts before applying these findings to their settings. For example, a country may have similar cadres of health workers but their roles or training may be different than what has been presented here for Peru and/or Uganda.

### Placing this study in the literature

Context has been found to be important in developing and implementing guidelines to bridge the gap between research evidence and its practical use [24, 25]. A study by Gagliardi et al. [26] showed that one of the preferred approaches of clinical guideline developers was to include information within the guideline that would help users implement it. One example is that specific and actionable recommendations are more likely to be implemented [27]. Wang, Norris and Bero [28] have further highlighted the need for WHO guidelines to improve their implementation strategies. While WHO produces general guidance, it is up to each jurisdiction to tailor these recommendations to fit their own needs and realities. Contextualization is one step in the shaping of global recommendations to make them more specific and actionable to these settings. The workbook adds to this emerging field by supporting users in contextualizing guidance by addressing health system arrangements and political system considerations in policy development and implementation. This feature of the workbook was well received by participants.

### Implications for policy and practice

There are four implications for policy and practice found through this study. First, the workbook was useful for contextualizing global guidance in two, quite different, settings. Organizations that produce global guidance, such as WHO, need to consider institutionalizing the application of workbooks (or other tools) into their guidance development processes to support the contextualization of each guidance document. Second, it is important to address the expectations of this work and to think broadly about the role of WHO in creating and contextualizing health systems guidance, as neither guidance documents nor workbooks (or other similar tools) could address all possible scenarios to account for the specific contexts of each country. Consideration could be given to having WHO regional offices and country offices support the development of regional or national guidance to further contextualize recommendations. Third, while the workbook may simplify this process by providing a systematic tool, it cannot replace the work required by a team of methods and content experts who understand the health system and the priorities of the government or Ministry of Health. It seems this would be an important consideration for countries that are looking to establish an EVIPNet or that have not already incorporated these country experts into their current processes. Lastly, some low- and middle-income countries may have limited capacity for local health and political system analysis and for embedding this work into their policy-making processes. WHO and partner organizations may have a role to play in helping countries build this capacity.

### Implications for research

There are three implications for research that were found. First, how contextual factors and components of the processes found in this study affect ultimate policy decisions and their implementation is not known. Empirically analysing these various stages, while potentially lengthy, will be needed to understand the implications of the various factors. Setting up an inventory of briefs and policies arising from global guidance may be useful in this endeavour. Second, the process of using the workbook evaluated the OptimizeMNH guidance as well as revealing gaps in data. Feedback mechanisms need to be established so that these findings can be considered during guidance development and during research priority-setting processes. Establishing and evaluating possible feedback mechanisms could be a fruitful area of study. Third, evaluating how the workbook was used in this process provided feedback on the workbook itself and ways to improve it. Further empirical work testing the workbook would tailor this tool for its use in various settings and for different topics.

## Conclusion

This study evaluates the use of a workbook to support the contextualization of WHO’s OptimizeMNH guidance in two countries — Peru and Uganda. Findings surrounding country-level contextual factors, steps in the process of using the workbook and evaluation of the workbook are presented. Organizations that produce global guidance, such as WHO, need to consider institutionalizing the application of the workbook into their guidance development processes to help users at the national or subnational levels create actionable and context-relevant policies. Feedback mechanisms also need to be established so that the evidence briefs and health policies arising from global guidance are tracked and the findings coming out of such guideline contextualization processes can be considered during future guidance development and research priority-setting.

## Supplementary information

**Additional file 1.** Documents reviewed for the study.

**Additional file 2.** Select demographic, social, economic, epidemiological and health system contextual factors for Peru and Uganda.

## Data Availability

The datasets (interview transcriptions, documents and reflexive journal) used during the current study are available from the corresponding author on reasonable request.
