# Supplementary material for: Developing evidence briefs for policy: a qualitative case study comparing the process of using a guidance-contextualization workbook in Peru and Uganda
Source: Health Res Policy Syst. 2019 Nov 21;17:89. doi: 10.1186/s12961-019-0488-0 (PMC6868683; doi:10.1186/s12961-019-0488-0)
Supplement: Supplementary file 1 — Additional file 1. Documents reviewed for the study. [file 12961_2019_488_MOESM1_ESM.docx]

Additional file 1. Documents reviewed for the study

| **Date(s)** | **Document Title** | **Document Type** | **Source** | **Author** |
| --- | --- | --- | --- | --- |
| Revised 2001 | Health services system profile of Peru | Document | Internet search | PAHO |
| 2005 | Norma técnica de planificación familiar | Document | Personal email | MINSA |
| 2009 | Uganda’s health care system explained | Document | Internet search | Kavuma |
| 2010 | An evidence-based policy brief: Task shifting to optimise the roles of health workers to improve the delivery of maternal and child healthcare | Document | Personal email | Nabudere, Asiimwe & Mijumbi |
| 2011 (Updated) | SURE guides for preparing and using evidence-based evidence briefs | Website | Interviewee | WHO / SURE |
| 2012 | OptimizeMNH: Optimizing health worker roles to improve access to key maternal and newborn health interventions through task shifting | Document | Interviewee | WHO |
| 2012 | Annex 8. Contextualizing the guidelines - workbook | Document | Principal investigator, online search | WHO |
| 2012 | Encuesta a Establecimientos de Salud con Funciones Obstétricas y Neonatales. ENESA 2009-2012 | Document | Personal email | Instituto Nacional de Estadística e Informática |
| 2012 | Encuesta Demográfica y de Salud Familiar – ENDES | Document | Personal email | Instituto Nacional de Estadística e Informática |
| 2012 | Uganda Health System Assessment | Document | Internet search | Ministry of Health, Health Systems 20/20, & and Makerere School of Public Health |
| 2012 | Uganda Demographic and Health Survey | Document | Internet search | Uganda Bureau of Statistics (UBOS) and ICF International Inc. |
| 2013, Jun | Nota técnica (Peru) – Interventions to increase institutional delivery through optimizing health worker roles following WHO’s 2012 recommendations (Intervenciones dirigidas a fortalecer el acceso al parto institucional a través de la optimización de las funciones del trabajador de salud empleando la guía de recomendaciones de la Organización mundial de la Salud de 2012)  Only final draft listed, but multiple earlier drafts and parts of drafts reviewed | Document | Personal email | Secretariat for evidence brief in Peru |
| 2013 | UNICEF – Statistics, Peru | Website | Internet search | Unicef |
| 2013 | UNICEF – Statistics, Uganda | Website | Internet search | Unicef |
| 2014 | WHO - Peru | Website | Internet search | WHO |
| 2014 | WHO - Uganda | Website | Internet search | WHO |
| 2014 | Policy briefs - SURE | Website | Internet search | WHO |
| n.d. | The world factbook - Peru | Website | Internet search | CIA |
| n.d. | The world factbook - Uganda | Website | Internet search | CIA |
| 2014, Mar 13 | Letter from Peru’s Ministry of Health (MINSA) to Peru’s National Institute of Health (INS) on priorities | Document | Personal email | MINSA |
| 2014, May 28 | Feedback on draft evidence brief | Document | Personal files | Principal investigator |
| 2013, Apr | Health in the Americas – Peru | Website | Internet search | PAHO |
| 2014, Jun 18 | Cover letter for working group meeting (Uganda) | Document | Personal files | Secretariat for evidence brief in Uganda |
| 2014, Jun 18 | Agenda for working group meeting (Uganda) | Document | Personal files | Secretariat for evidence brief in Uganda |
| 2014, Jun 18 | Power point presentation for working group meeting (Uganda) | Document | Personal files | Secretariat for evidence brief in Uganda |
| 2014, Jun 18 | Terms of reference for evidence brief in Uganda (Uganda) - Working paper – most updated draft listed, but multiple earlier drafts and parts of drafts reviewed | Document | Personal files | Secretariat for evidence brief in Uganda |
| 2014, Jul 8 | Timeline for work in Uganda - Working paper – most updated draft listed, but multiple earlier drafts and parts of drafts reviewed | Document | Personal files | Secretariat for evidence brief in Uganda |
| 2014, Jul 18 | Nota técnica (Peru) – Interventions to decrease barriers in accessing modern family planning methods at the regional level (Intervenciones dirigidas para disminuir las limitaciones de acceso a métodos modernos de planificación familiar a nivel regional) - Working paper – most updated draft listed, but multiple earlier drafts and parts of drafts reviewed | Document | Personal email | Secretariat for evidence brief in Peru |
| 2014, Aug 20 | Letter of information for health workers (Uganda) | Document | Personal files | Secretariat for evidence brief in Uganda |
| 2014, Oct 20 | Report from health centres (Uganda) | Document | Personal email | Research Assistant |
| 2015 | Evidence briefs / issue briefs | Website | Internet search | McMaster Health Forum |
| 2014, May 2  2014, May 28  2014, Jun 20  2014, Jul 30 | Meeting notes / Participant-observations – Peru – multiple:  Gmail chat transcription with Secretariat  Skype meeting with Secretariat  Gmail chat transcription with Secretariat  Skype meeting with Secretariat | Documents | Participant-observations | Principal investigator / Secretariat for evidence brief in Peru |
| 2013, Oct 24  2013, Dec 19  2014, Apr 30  2014, May 8  2014, May 15  2014, Jun 10  2014, Jun 13  2014, Jun 16  2014, Jun 18  2014, Jun 19  2014, Jun 20  2014, Jul 8  2014, Aug 1  2014, Aug 13  2014, Nov 5 | Meeting notes / Participant-observations – Uganda – multiple:  Skype meeting with contact in Uganda  Notes from meeting of Uganda team  Skype meeting with Secretariat  Skype meeting with Secretariat  Skype meeting with Secretariat  Guidance development session  Guidance development session  Guidance development session  Guidance development session  Guidance development session  Guidance development session  Skype meeting with Secretariat  Skype meeting with Secretariat  Skype meeting with Secretariat  Skype meeting with Secretariat | Documents | Participant-observations | Principal investigator / Secretariat for evidence brief in Uganda |
| 2012, Mar 3 –  2013, Nov 19 | Reflexive Journal I (Workbook) | Reflexive Journal –  195 pgs, | Principal investigator | Principal investigator |
| 2013, Nov 20 –  2014, Jun | Reflexive Journal II (Workbook) | Reflexive Journal –  127 pgs | Principal investigator | Principal investigator |
| 2014, June 5 –  2015, Jan 23 (ongoing) | Reflexive Journal III (Workbook) | Reflexive Journal –  93 pgs | Principal investigator | Principal investigator |
| 2012-2014 |  | Personal correspondence | Personal emails | Multiple |

Does not include documents reviewed for developing the evidence briefs
