# Supplementary material for: Developing evidence briefs for policy: a qualitative case study comparing the process of using a guidance-contextualization workbook in Peru and Uganda
Source: Health Res Policy Syst. 2019 Nov 21;17:89. doi: 10.1186/s12961-019-0488-0 (PMC6868683; doi:10.1186/s12961-019-0488-0)
Supplement: Supplementary file 2 — Additional file 2. Select demographic, social, economic, epidemiological and health system contextual factors for Peru and Uganda. [file 12961_2019_488_MOESM2_ESM.docx]

Additional file 2. Select demographic, social, economic, epidemiological, and health system contextual factors for Peru and Uganda

| **Contextual factors** | **Peru** | **Uganda** | **Comments** | **Sources of information** |
| --- | --- | --- | --- | --- |
| Total area | 1,285,216 sq km | 241,038 sq km | Peru is 5 times larger than Uganda | Central Intelligence Agency, n.d.a., n.d.b |
| Administrative divisions | 25 regions and 1 province (Lima) | 112 districts | Uganda has over 4 times the administrative divisions of Peru | PAHO, 2012;  Uganda Bureau of Statistics (UBOS) and ICF International Inc, 2012 |
| Total population (2012) | 29,988,000 | **36,346,000** | Relatively similar population | WHO, 2014a, 2014b |
| Urbanized population (%), 2012 | 78 | 16 | Delivery of services is greatly impacted by where people live | Unicef, 2013a, 2013b |
| Birth registration (%), 2005-12, urban/rural | 96/94 | 38/29 | Monitoring and evaluation activities need to reflect differences in reporting and account for where and how measurements are made (e.g., community level vs. national level, self-reported vs. household survey) | Unicef, 2013a, 2013b |
| Gross National Income per capita (PPP USD $, 2012) | 10,240 | 1,140 | Peru’s GNI per capita is 9 times greater than Uganda’s | Unicef, 2013a, 2013b |
| Total expenditure on health per capita (Intl $, 2012) | 555 | 108 | Peru’s total expenditure on health per capita is 5 times greater than Uganda’s | WHO, 2014a, 2014b |
| Total expenditure on health as % of Gross Domestic Product (2012) | 5.1 | 8.0 | Uganda spends more on health as a percent of its GDP | WHO, 2014a, 2014b |
| Life expectancy at birth m/f (years, 2012) | 75/79  life expectancy ranges from 72-77 years for Lima, Callao, and certain coastal departments, to 57-63 years for the Andean departments | 56/58 | People in Peru have a life expectancy of 20 years over that of people in Uganda. However, variations exist within countries as well. | WHO, 2014a, 2014b; PAHO, 2001 |
| Adult HIV prevalence (%), 2012 | 0.4 | 7.2 | HIV prevalence affected the recommendations regarding external cephalic version (ECV) in Uganda | Unicef, 2013a, 2013b |
| Total fertility rate (TFR), 2012 | 2.4  2.3 for urban areas and 4.6 for rural areas;  variance depending on the educational level of women, with TFRs of 6.9 for women  without education, 5.0 for women with primary education, 3.0 for women with secondary education, and 2.1 for women with higher education | 6.0 | Uganda has a higher total fertility rate than Peru, but wide variation exists within Peru based on urban/rural dwelling and women’s educational level. These are important considerations for equity. | Unicef, 2013a, 2013b:  PAHO, 2001 |
| Births with skilled attendant present (%), 2008-2012 | 87 | 57 | Having a skilled attendant at birth is one of the most important ways to decrease maternal mortality. Improvements in this indicator need to consider cultural preferences and the urban/rural distribution of the population and of health human resources. | Unicef, 2013a, 2013b |
| Lifetime risk of maternal death (2010) | 1 in 570  More than 50% of maternal deaths are concentrated in eight regions: Cajamarca, Puno, La Libertad, Loreto, Piura, Junín, Huánuco, and Cusco | 1 in 49 | 1 in 5,200 in Canada | Unicef, 2013a, 2013b  PAHO, 2012 |
| Under-5 mortality rate, 2012 | 18 | 69 | 4 times greater in Uganda | Unicef, 2013a, 2013b |
| Infant mortality rate (under 1), 2012 | 14 | 45 | 3 times greater in Uganda | Unicef, 2013a, 2013b |
| Neonatal mortality rate, 2012 | 9 | 23 | 3 times greater in Uganda | Unicef, 2013a, 2013b |
| **Health system** |  |  |  |  |
| Governance arrangements | The Ministry of Health is responsible for policy in the health sector. Each region is responsible for modifying policy to fit its needs and implementing policies and programs. | The Government of Uganda, through the Ministry of Health (MoH), is responsible for developing policies and frameworks for delivering health services. Either the MoH, or parliament, can initiate policies. Multiple players, including other ministries and outside donors, contribute to health policy. Uganda has a decentralized system in which power, authority and resources are distributed from the central government to the districts. However, some of the districts lack the personnel, resources and training to carry out their duties. |  | Observations;  PAHO, 2001;  PAHO, 2012;  Ministry of Health, Health Systems 20/20, & and Makerere School of Public Health, 2012); |
| Financial arrangements | Mix of public and private financing. Each region is allocated a budget from the central Ministry and is accountable for finances regarding health. Three types of health insurance exist: social security (ESSALUD), the Armed Forces and National Police, and private insurance. Donor financing is about 2% of total health expenditure. | Mix of public, private, and donor-funded (32-over 50%) financing. Each region is allocated resources from the central government but resources are often not enough to support implementation of policy and programs. | Regulations within the public sector and the private sector need to be considered. Also, the role of NGOs cannot be discounted in the policymaking process. |  |
| Delivery arrangements | The public sector is made up of the Ministry of Health, ESSALUD (Social Security), and the services of the Armed Forces and the National Police. The public subsector has 51% of total hospitals, 69% of health centers, and 99% of health posts, located in remote rural areas and marginal urban areas.  The private subsector mainly operates in the larger cities and is made up of clinics, physician’s offices, and NGOs. | Service delivery is provided by public and private sectors (about 50% each). The public sector includes Village Health teams (or Health Centre I – HCI) within the communities and tiered levels of care at the district level (HCII, HCIII, and HCIV) up to general hospitals (formerly district hospitals). The regional and national hospitals are semi-autonomous, while the district health services and general hospitals are managed by local governments.  Private health service providers comprise private not-for-profit organizations, private for-profit health care providers (or commercial health care providers), and traditional and complementary medicine practitioners.  Staffing of these facilities are variable, especially at the HCII level where there are “fill rates, ranging from 0 percent to well over 100 percent. The 2011 Audit Report noted that “most of the HC IIs have the Nursing Assistants/Aides as the in-charges which compromises the quality of care provided at these service delivery points” | There is poor coordination between the public and private systems in both countries |  |
| **Capacity for knowledge translation and evidence-informed policymaking** |  |  |  |  |
| EVIPNet (Evidence-informed policy network) equivalent | The unit for analysis and evidence production for public health (UNAGESP) within the National Institute of Health (INS), which supports the Ministry of Health (MINSA) in using evidence in policymaking. | - The Region of East Africa Community Health (REACH) Policy Initiative set up years ago between multiple countries in East Africa to help use evidence in developing policy.  - SURE project (Supporting the use of research evidence for policy in African health systems) lasted for five years and was funded and supported through the European Commission’s 7th Framework Programme to develop and evaluate knowledge translation strategies. | Both countries have formal processes in place to support evidence-informed policymaking. Peru’s structure is more formalized as it has direct links with the Ministry of Health through its directives and funding sources. Uganda has more informal channels to the Ministry and usually reaches policymakers at the Ministry through professional contacts and involvement of these contacts in the working groups for developing evidence briefs. | Interviews, SURE and REACH websites |
| Complementary initiatives focused on health system issues | None found | - Rapid response system started in Uganda under SURE project and is now being adapted by other countries  - Uganda Clearinghouse for Health Policy and Systems Research  - Africa Centre for Systematic Reviews and Knowledge Translation |  |  |
| Affiliation with academic centres | No | Yes – based within Makerere University in Uganda with linkages to universities in Canada and Norway | Affiliation with academic centres often grants people access to sources of evidence, such as research databases. This is an important consideration for the capacity to find and use evidence in decision-making. |  |
| Number of prior evidence briefs and rapid response reports | 2 | 5 evidence briefs and 71 rapid response reports (as of January 22, 2015 – personal correspondence) under the SURE project.  Uganda completed an evidence brief on a similar topic in 2010, prior to the release of the OptimizeMNH guidance and the workbook | Peru has less experience in developing evidence briefs than Uganda. |  |
| **Usual process for developing evidence briefs and holding policy dialogues** |  |  |  |  |
| Selecting a topic | 1) A topic is brought forward by the Ministry of Health or  2) A need is determined by an expert (e.g., the case of micronutrient powders in Peru) | 1) Priority-setting process under the SURE project involving interviews with stakeholders and determining priorities or 2) Policymaker asks for evidence on a particular topic for a rapid response |  | Interviews, observations |
| Developing an evidence brief | One or several people draft the evidence brief (equivalent to evidence brief) by using evidence to define the topic, develop policy options and look at implementation considerations. This work is presented to the Ministry to ensure it meets their needs and to keep them aware of the work. | A team of authors determine a search strategy and develop terms of reference section-by-section (clarify the problem, develop policy options and identify implementation considerations). The authors revise each section based on feedback from the working group and use the terms of reference to write the evidence brief. |  |  |
| Using guides in the development of evidence briefs | Worksheets, developed by the Pan-American Health Organization (PAHO) and which are similar to the SURE guides, are followed to develop the evidence brief. | The SURE guides are used to develop the sections of the terms of reference or to act as a checklist to ensure all items have been considered for the evidence brief. |  |  |
| Involving experts, policymakers, stakeholders and researchers | 1) May be included in the development of the evidence brief, depending on the topic, but mainly occurs during the policy dialogue.  2) Written feedback is concurrently obtained from experts who are not in attendance at the policy dialogue | 1) A working group made up of policymakers, stakeholders and researchers provides input throughout the process.  2) The brief is sent out to 3-4 external reviewers (academics and policy practitioners and implementers) and feedback is incorporated prior to the policy dialogue  3) Experts, policymakers, stakeholders and researchers are included in the policy dialogue |  |  |
| Convening a policy dialogue | 30-40 policymakers, stakeholders, representatives of groups and researchers discuss the implications of the evidence brief and provide their opinions and tacit knowledge. A series of dialogues may be held and recommendations are made. | 15-20 policymakers, stakeholders and researchers discuss the implications of the brief and refine the work based on the larger groups’ perspectives and knowledge, and to sensitize the group on this particular problem and potential solutions. |  |  |
| Making policy recommendations | The Ministry of Health then decides whether to adopt the recommendations, and, if adopted, supports the implementation of the recommendations | Usually, a champion is sought during the policy dialogue to move the work forward within policymaking circles |  |  |
| Time to develop evidence brief | 4-6 months | 6-8 months |  |  |
